# Eye-Tracking Analysis for Emotion Recognition

**DOI:** 10.1155/2020/2909267

**Published:** 2020-08-27

**Authors:** Paweł Tarnowski, Marcin Kołodziej, Andrzej Majkowski, Remigiusz Jan Rak

**Affiliations:** Institute of Theory of Electrical Engineering, Measurement, and Information Systems, Warsaw University of Technology, Warsaw 00-662, Poland

## Abstract

This article reports the results of the study related to emotion recognition by using eye-tracking. Emotions were evoked by presenting a dynamic movie material in the form of 21 video fragments. Eye-tracking signals recorded from 30 participants were used to calculate 18 features associated with eye movements (fixations and saccades) and pupil diameter. To ensure that the features were related to emotions, we investigated the influence of luminance and the dynamics of the presented movies. Three classes of emotions were considered: high arousal and low valence, low arousal and moderate valence, and high arousal and high valence. A maximum of 80% classification accuracy was obtained using the support vector machine (SVM) classifier and leave-one-subject-out validation method.

## 1. Introduction

### 1.1. Motivation

Emotions play a very important role in everyone's life. People feel emotions during everyday tasks, during interpersonal communication, when making decisions, learning, or during cognitive activities. For several years, a large increase in the number of studies on methods of automatic recognition of emotions has been observed. This is due to the increasing number and widespread use of electronic devices such as smartphones, tablets, and computers. The issue of recognizing emotions is an interdisciplinary problem and includes computer science, psychology, and cognitive science. The development of effective methods for recognizing emotions can not only improve the interaction of people with machines, but also contribute to the development of other areas such as psychology, medicine [1], education [2], and entertainment [3].

### 1.2. State of the Art

Automatic recognition of emotions in medicine can be used to diagnose and treat diseases such as posttraumatic stress disorder [4] and depression [5]. It is also used in diseases such as autism or Asperger's syndrome [6, 7]. Another application of emotion recognition is education and supporting distance learning. In [8], a speech signal was used to recognize students' emotions during the lesson. In [9], a tool is presented to visualize the degree of attention and involvement of students, using the measurement of electrodermal skin activity. Emotion recognition systems can improve teacher-student interaction and, as a result, lead to better learning results. Currently, due to the rapidly growing number of multimedia materials appearing on the Internet, numerous studies are carried out related to their automatic labeling. Movies or music is often designed to evoke specific emotions; hence the automatically generated labels should contain information about the emotional characteristics of the material [10]. In [11], an EEG signal was used to label movies in terms of evoked emotions. To recognize emotions, the features of the signal registered for a group of people watching the same set of movies were used. Similar studies are described in [12, 13] using, in addition to the EEG signal, facial expressions.

Emotions are felt by a person as certain reactions of the body to a given situation or stimulus. The occurrence of these reactions is the basis for the operation of systems for recognizing the emotional state of a person. Facial expressions [12, 14, 15], speech signal recordings [16, 17], and brain activity and changes in other human physiological parameters are subject to testing. In the case of brain activity, signals from the central nervous system are recorded using electroencephalography (EEG) [12, 14, 15], as well as medical magnetic resonance imaging (fMRI) [18]. Other physiological signals used include: electrocardiography (ECG) [19–21], electromyography (EMG) [22], electrodermal activity (EDA) [19, 20, 23], heart rate [24–26], respiration rate and depth [24, 27], and arterial pressure [24]. Eye-tracking [28–32] and pupil width [33–36] are also used to recognize emotions.

Interest in eye movement research dates back to the nineteenth century when the method of direct observation was used. Then, invasive eye-trackers were constructed using a special type of lens placed on the eyeball. At present, noncontact devices, which use infrared cameras, are most commonly used for eye-tracking. The development of such devices increased the popularity of eye movement research among scientists from many different fields. Eye-tracking research is applied in areas such as cognitive psychology, neuropsychology, usability testing, or marketing [37]. The eyeball movement is examined while viewing the presented materials, e.g., photos or movies, and while reading, playing, browsing the web, or using graphical user interfaces. Few attempts have also been made to use eye-tracking to recognize emotions [32]. In [28], features associated with eye movements were used to recognize three classes of emotions. The classification accuracy obtained was 77.8%. In [38], for three classes of emotions recognized using eye-tracking methods, the classification accuracy was 68.8%. In [30], the use of features such as fixation duration, amplitude and duration of saccades, and blinking frequency for classification of four classes of emotions was described, achieving 67.82% accuracy. In all mentioned studies, emotions were evoked by presenting movies.

The eye-tracking often includes measuring the diameter of the pupil, which is responsible for regulating the amount of light falling on the retina. In addition to light intensity, the pupil response is also influenced by other factors. These factors include the emotions experienced by oneself [39, 40]. To date, studies have shown that the pupil diameter increases when feeling excited [34, 36]. In [29], an attempt to recognize three classes of emotions (positive, negative, and neutral) evoked by movies using pupil width was presented. Classification accuracy of 58.9% was achieved. The study [41] describes research on recognizing emotions caused by playing a video game. A set of features related to pupil width were used for the classification. For three classes of emotions, the classification accuracy achieved was 76% and 61.4%, respectively, for the arousal and the valence scales. The work [35] presents the use of pupillometry to measure emotional arousal in videos.

### 1.3. The Aim of This Paper

The purpose of the presented research is to analyze whether it is possible to recognize emotions by using eye-tracking signal. Emotions have been caused by the presentation of video material. For the present research, an experiment was designed during which a group of 30 participants watched a set of 21 fragments of movies intended to evoke emotions. During movie presentation, eye-tracking data were recorded. As features, we used eye-movement specific elements such as fixations and saccades. Features related to the pupil diameter were also calculated. We recognized three classes of emotions: high arousal and low valence, low arousal and moderate valence, and high arousal and high valence. Three classifiers were tested: SVM, LDA, and *k*-NN. The leave-one-subject-out method was used to assess the quality of the classification. Individual, successive stages of the research were the following:

- acquisition of eye-tracking data while evoking emotions
- performing signal preprocessing
- removal of the effect of luminance on pupil width
- calculation of eye-tracking features related to eye movements and pupil width
- classification of emotions using SVM, LDA, and *k*-NN

Video material is characterized by fast scene changes and moving objects. The dynamics of the movies can greatly influence the eye-tracking features and the possibility to use them for emotion recognition. Innovative in our research is that we examined the effects of the dynamics of movies on classification accuracy. Further, when measuring the pupil diameter, the impact of luminance and lighting conditions is very important. The effect of luminance on pupil width was removed using linear regression.

## 2. Materials and Methods

### 2.1. Evoking Emotions

An important element of the study was evoking emotions in participants of the experiment. Emotions are most often caused by the presentation of visual stimuli (images and movies) [34, 38, 42, 43] and sound stimuli (music and sounds) [36, 44]. In our experiment, emotions were evoked by a video presentation on a monitor screen. The presented material comprised short movies along with the soundtrack. Twenty-one movies were used in the experiment. They elicited six basic emotions, as defined in [45], that is, happiness, sadness, anger, surprise, disgust, and fear. Several other movies were selected to be as neutral as possible and did not cause any of the above emotions. The selection of movies that cause a specific emotion is a difficult task. Therefore, a survey was conducted to ensure that a movie affects the examined participant in the intended way. The questionnaire was designed to examine the feelings and emotions experienced after seeing individual pieces of movies. Each participant was asked the following 3 questions about each movie:

1. Evaluate the emotions you experienced while watching a movie from 1 (unpleasant-low valence) to 9 (pleasant-high valence).
2. Rate the intensity of the emotions you experienced while watching a movie from 1 (nonstimulating-low arousal) to 9 (highly stimulating-high arousal).
3. Choose the type of emotion you felt while watching the movie: neutral, happiness, sadness, anger, surprise, disgust, and fear. A participant also had the opportunity to describe his/her feelings.

**Figure 1:**
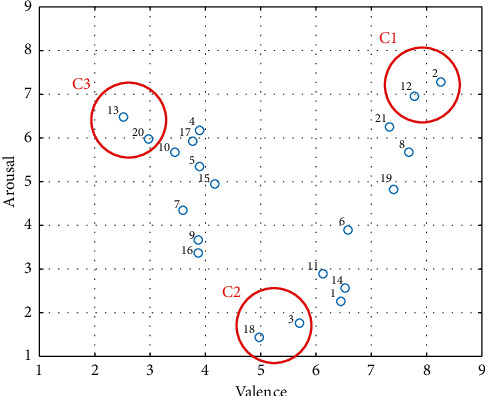
Distribution of emotions caused by movies (numbered from 1 to 21) on the valence-arousal plane along with created classes (marked red).

The survey among the participants allowed creating a circumplex model of emotions [46, 47]. In this model, the horizontal axis represents emotion valence, whereas the vertical axis represents arousal. Each emotion can be considered as a linear combination of these dimensions.  Figure 1 shows the distribution of emotions caused by each movie (numbered from 1 to 21) used in the experiment on the valence-arousal plane. The distribution is based on the answers given by the participants in surveys.

The distribution of emotions resembles the shape of letter “V;” this is because movies that are very pleasant or very unpleasant are rated as stimulating at the same time. Neutral materials are generally rated as nonstimulating [48]. These tendencies are also reported in previous works [49, 50]. We created three classes of emotions from the distribution of movies on the plane. Each class included two movies. Class C1 covered videos that were rated in surveys as very pleasant and very stimulating (high arousal and high valence). Class C2 included neutral, low stimulating movies (low arousal and moderate valence). Class C3 contained very unpleasant and highly stimulating movies (high arousal and high valence). Only these 6 movies were further considered in the experiment.  Table 1 presents the mean and median values of valence and arousal assigned to the selected movies by the participants. Movies #2 and #12 presented dancing women with rhythmic music. Movie #3 presented a bird's eye view of the highway with calm music. Movie #18 presented the weather forecast. Movie #13 included scenes of violence against a woman, while movie #20 showed amputation of a finger.

### 2.2. Participants of the Experiment

A specific group of people selected based on age, gender, and education were invited to participate in the experiment. Participants were recruited through an advertisement on the website of Faculty of Electrical Engineering, Warsaw University of Technology. Finally, 30 volunteers took part in the experiment. All participants were male third-year students with an average age of 21.25 ± 0.74 years. They were informed about the overall purpose and organization of the experiment and agreed to participate in it. Volunteers were not paid for participating in the experiment.

### 2.3. Experimental Procedure

**Figure 2:**
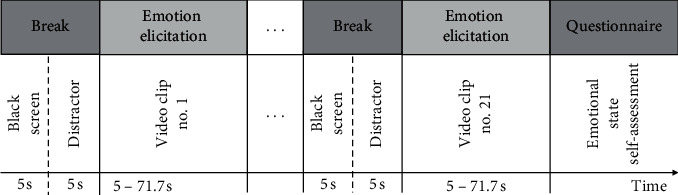
Typical sequence of the presented movies.

The experiment was carried out in a specially prepared room, and its temperature and lighting were the same for all participants. The test stand consisted of a PC, EyeTribe eye-tracker, and two Creative Inspire T10 speakers. The experiment always started in the morning between 7:00 and 8:00 AM. The participants were asked to take a comfortable position on the armchair that supported their hands and back in front of the computer monitor. Next, eye-tracker calibration was performed. The person supervising the experiment then left the room, and the participant alone watched the video material. The movies shown had a soundtrack. The sound volume was identical for all participants. We developed our own software responsible for controlling the course of the experiment, which enabled simultaneous display of movies and eye-tracking signal acquisition [51]. The movie prepared for the experiment lasted 8 min 10 s. Different clips of the video material that evoked different emotions were separated by a sequence of 5 s of black screen and 5 s of colour animated curves that resembled a screen saver. The break between the videos was intended to curtail the previous emotions and prepare the participant for the next part of the movie. Each participant watched the same video material with a random order of 21 short movies associated with particular emotions.  Figure 2 shows a typical sequence of the presented movies. After the video material was played, each participant had to answer the questions in a survey on emotions caused by movies.

### 2.4. Acquisition of Eye-Tracking Data

The EyeTribe [52] optical eye-tracker was used to register oculographic signal. The EyeTribe calculates a pair of (*X*, *Y*) coordinates of the user's gaze point with respect to a screen the user is looking at. The coordinates are expressed in pixels. Accuracy of calculating user's eye gaze coordinates is approximately 1°. Such an accuracy can be obtained after proper calibration of the device. Calibration using 9 measurement points was performed for each participant. The EyeTribe also provides information about the occurrence of eye fixations and measures the pupil diameter of both eyes. The eye-tracking signal was registered with a frequency of 60 Hz.

### 2.5. Signal Preprocessing

**Figure 3:**
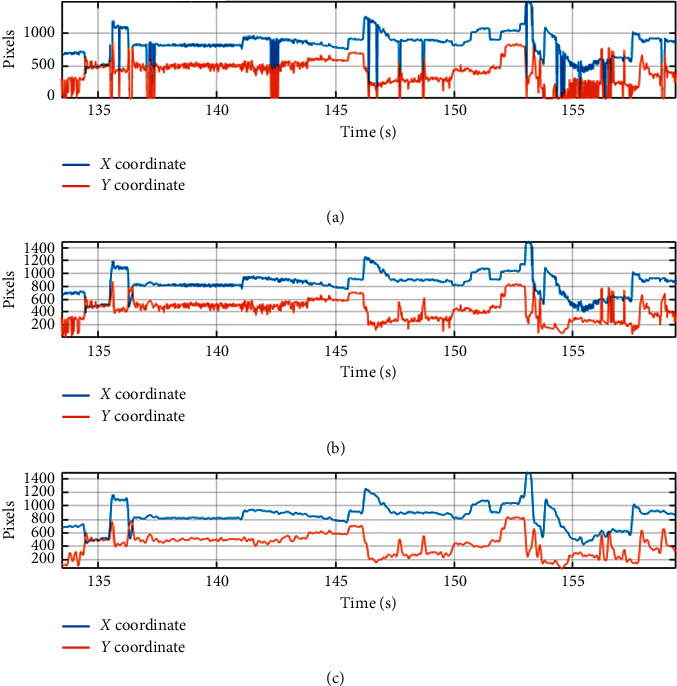
Preprocessing of eye-tracking signal: (a) pure signal, (b) signal after interpolation, and (c) signal after low-pass filtering.

Some of the participants' eye movement information was lost, for example, during blinking or during other eye or head movements. Data from a participant were rejected completely when the number of lost samples was greater than 25%. Thus, data recorded for 5 participants were rejected. Further analysis was performed on data recorded for the remaining 25 participants. Samples, lost due to, for example, blinking, were supplemented by linear interpolation. The signal was also filtered with 4^th^ order low-pass Butterworth filter with a cutoff frequency equal to 5 Hz. Filtration was designed to eliminate high-frequency noise.  Figure 3(a) shows the recorded eye-tracking signal (*X* and *Y* coordinates of the gaze point expressed in pixels): (B) interpolated signal and (C) signal after low-pass filtering.

### 2.6. Feature Extraction

**Figure 4:**
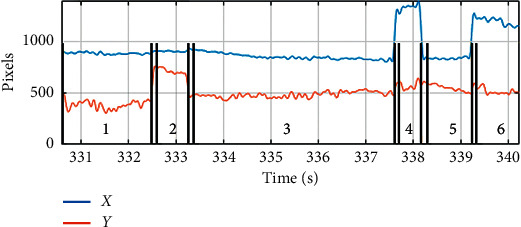
A fragment of an eye-tracking (fixations, saccades, and coordinates of the “sight”).

The analysis of eye-tracking data involved calculating the features of the signal that depends on the emotional content of a movie. Raw data points were processed into eye movements such as fixations and saccades [37]. There are many algorithms to calculate fixation; these algorithms are based on the speed of eye movement (I-VT) and data scattering (I-DT) or are related to areas of interest (AOI-I) [53]. The EyeTribe eye-tracker has a built-in algorithm that classifies a given sample of a registered signal and informs one whether a given signal sample represents a fixation.  Figure 4 shows a fragment of the registered visual coordinates along with the marked fixations. There are 6 fixations identified by the eye-tracker in the shown signal fragment.

Features were calculated in time windows covering each of the movie fragments. Fixations were used to calculate features such as the number of fixations, mean value, variance, skewness, and kurtosis of duration of fixations. The fixation durations were expressed as the number of samples. Based on fixations, the overall fixation vector (OFV) was also calculated. This feature considers the number, position, and duration of fixations. It is described by the following [37, 54]:

(1) $$\begin{array}{c} \text{OFV}=\sum_{i=1}^{N}t_{i}v_{i}, \end{array}$$

where **v**_**i**_=(*x*_*i*_ − *x*_*c*_, *y*_*i*_ − *y*_*c*_) is a vector with an origin in the center of the screen (*x*_*c*_, *y*_*c*_) and the end at the fixation point (*x*_*i*_, *y*_*i*_), *t*_*i*_ is the duration of the *i*-th fixation. Pairs (*x*_*c*_,  *y*_*c*_) and (*x*_*i*_, *y*_*i*_) are coordinates, expressed as pixels, of the center of the screen and the fixation point, respectively. Mean value, variance, skewness, and kurtosis of amplitudes and the duration of the saccades were also calculated. The duration of saccades was expressed as the number of samples, while amplitude was expressed as pixels. Some features such as the number of fixations depend on the duration of the movie for which they were calculated. To normalize these features, they were divided by the length of the movie expressed as the number of samples. Therefore, these features were not correlated with the length of the analysed part of the movie.

The pupil diameter enables the detection of changes in the intensity of human emotions at a given moment [34, 36]. In addition to eye movement, the EyeTribe eye-tracker allows one to measure the pupil diameter. Mean value, variance, skewness, and kurtosis of the pupil diameter, which were measured during watching a given movie, were calculated.  Table 2 lists the calculated features. The features were grouped according to the type of eye movement.

### 2.7. Classification

The classification was performed using 18 eye-tracking features taken from 25 participants. The features were calculated for a whole movie associated with one type of emotion. Each emotion was evoked by two different movies. Features were standardized by centering them to have a mean value equal to zero and by scaling them to have standard deviation equal to one (*z*-score) for each participant. Therefore, for 25 participants, the total number of examples related to one emotion was 50 (2 movies × 25 volunteers). Hence, to classify emotions in pairs and in triplets, the number of examples was 100 and 150, respectively.

As classifiers, we used linear discriminant analysis (LDA), support vector machine with a square kernel (Quadratic SVM), and the nearest neighbour classifier (*k*-NN) with *k* = 11 [55]. The parameters of individual classifiers were selected experimentally based on the accuracy of the classification. Classification was performed in the user-independent mode. Training dataset included examples from 24 participants and testing dataset included examples from 1 remaining participant so leave-one-out method was used for tests. The division of the data into training and testing sets was repeated 25 times. This enabled calculation of the average classification accuracy. Examples that were used in the training process were not used again during testing the classifier.

## 3. Results

Classification was performed for three pairs and for all three classes of emotions together. Classification accuracies achieved for each case are shown in Table 3. The highest accuracy of 80% of the classification of three classes of emotions was obtained using the SVM classifier. When recognizing emotions in pairs, it turned out that the easiest is to distinguish between classes C1 and C2. On the other hand, the most difficult is to distinguish between classes C2 and C3. Tables 4–6 show the confusion matrixes for the classification of all three classes for SVM, LDA, and *k-*NN classifiers, respectively.

Then, for each classifier parameters, precision (P), recall (R), specificity (S), and F1-score (F1) were calculated for all three classes of emotions (Table 7).

Confusion matrixes analysis showed that the most classification errors appear for the C3 class. This is also confirmed by the values of precision, recall, and F1-score.

To answer the question of which features associated with eye movement can be a reliable indicator of emotions, statistical tests were performed. One-way analysis of variance (ANOVA) was used to determine whether features show a significant difference between the three classes. Before the ANOVA test was performed, it was checked whether the features satisfy normal distributions using Kolmogorov–Smirnov test. Distributions of the features were close to normal. Bonferroni post-hoc test for evaluating features among the groups was also made.  Table 8 presents the results of the ANOVA and Bonferroni tests for each feature. The Bonferroni test showed that 9 features are suitable for distinguishing between classes C1 and C2, 8 features for distinguishing between C1 and C3, and only 5 features for distinguishing between C2 and C3. This result is in line with previous observations; that is, the lowest classification accuracy was obtained when distinguishing between classes C2 and C3.

Mean values and standard deviations of all features for three classes of emotions are presented in Table 9.

**Figure 5:**
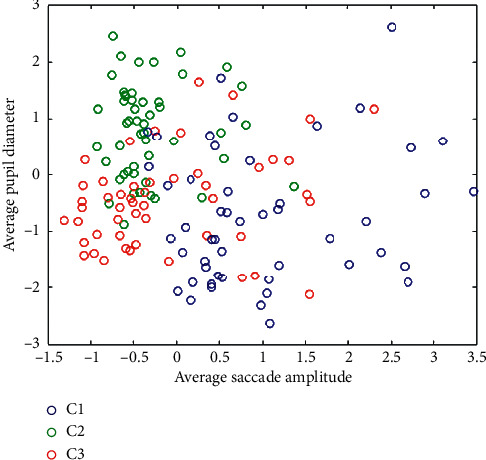
The distribution of the average pupil diameter and the average saccade amplitude for the three classes of emotions.

The participant's pupil diameter is one of the statistically significant features. The pupil diameter reached the smallest value when experiencing neutral emotions (low arousal). The average pupil diameters for C1, C2, and C3 classes were 0.85, -0.74, and -0.43 (z-score standardized), respectively. A similar trend was observed in other studies [28, 29, 34, 36].  Figure 5 shows the distribution of the two features with the smallest *p*-values (average pupil diameter and average saccade amplitude) for the three classes of emotions.

## 4. Discussion

**Figure 6:**
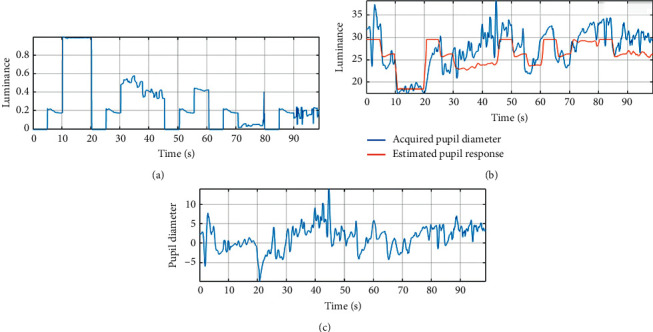
Removing the effect of movie luminance: (a) calculated luminance of a movie; (b) blue line, registered pupil diameter for a participant; red line, estimated pupil diameter (as a response for movie luminance); (c) pupil diameter after removing the luminance effect.

The obtained accuracy of emotion classification indicates that it is possible to distinguish emotions using eye-tracking. However, when using eye-tracking and pupil width, some additional factors that may affect the reliability of the results should be considered. The changes in the pupil diameter are largely dependent on the lighting conditions and the luminance of the movie [29, 56]. The effect of luminance is so large that it is impossible to use the pupil diameter directly to recognize emotions without prior processing it. We have assumed that pupil changes are partly due to changes in the luminance of the movie and partly due to the experienced emotions. Therefore, the effect caused by the luminance of the movie had to be removed. For this purpose, the luminance of the movie was assessed by calculating for each of its frames the V component in the HSV colour space. The process of removing the movie luminance effect from the measurements of the pupil diameter is presented in Figure 6.  Figure 6(a) shows a fragment of the luminance of the movie. Linear regression was used to model the relationship between the pupil diameter and luminance of a movie. For each participant, a linear model of the influence of luminance on the pupil diameter was calculated (coefficients *b*_0_, *b*_1_) using

(2) $$\begin{array}{c} \left[\begin{array}{c} y_{1}\\ y_{2}\\ \vdots \\ y_{n} \end{array}\right]=\left[\begin{array}{cc} 1 & x_{1}\\ 1 & x_{2}\\ \vdots & \vdots \\ 1 & x_{n} \end{array}\right]\left[\begin{array}{c} b_{0}\\ b_{1} \end{array}\right], \end{array}$$

where *y* is the average pupil diameter of both eyes recorded for a participant and *x* is the luminance value calculated for a movie frame.

Then, the estimated value of the pupil diameter *y*_est_ was calculated from

(3) $$\begin{array}{c} y_{\text{est}}=b_{0}+b_{1}x. \end{array}$$

This caused the pupil diameter to be independent of luminance changes ((4)). We assume that *y*_emo_ depends on the felt emotions.

(4) $$\begin{array}{c} y_{\text{emo}}=y-y_{\text{est}}. \end{array}$$

Figure 6(b) shows the registered pupil diameter value for participant S06 and its estimated (as a response for movie luminance) value *y*_est_=29.84 − 11.23*x*.  Figure 6(c) shows the pupil diameter of the same participant after removing the effect of luminance. The mean correlation between the pupil diameter and luminance for all volunteers was −0.49. This value was reduced to 0.006 after subtracting the estimated luminance effect.

**Figure 7:**
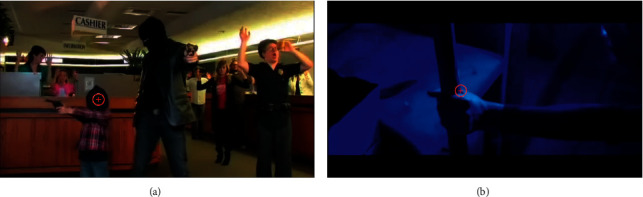
Two sample frames from movies with one participant's gaze positions.

We also carried out additional tests to assess whether the method of removing the influence of luminance is sufficiently effective. First, we tested the approach taken from a prior publication [23], in which neighbourhood of the gaze position was used to calculate the luminance, instead of the average luminance of the whole frame. The neighbourhood had a width of 2 degrees (of visual angle) around the gaze point, which corresponds to a circle with a diameter of 68 pixels (distance from the monitor 60 cm, resolution 1920 × 1080 pixels). Sample frames from movies with marked gaze positions are shown in Figure 7.

**Figure 8:**
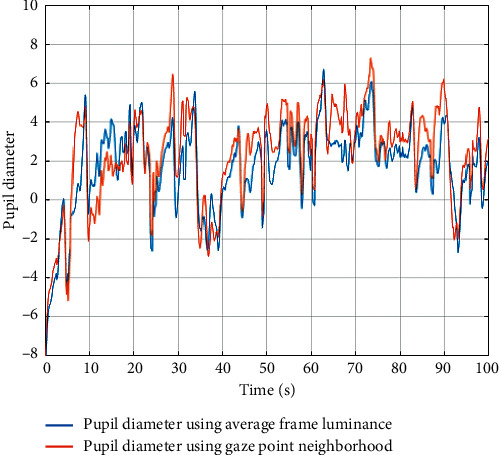
Changes in pupil eye diameter of one of the participants after removing the influence of luminance calculated using two methods.

The average frame's luminance was 0.4 for the left frame and 0.19 for the right one. The luminance in the neighbourhood of the gaze positions was 0.08 and 0.71, respectively. In both cases, the average luminance of the frame and the luminance of the neighbourhood of the gaze positions are significantly different from each other. In the frame on the left, the participant looked at one of the darker points, while, in the frame on the right, he looked at a bright point. One, however, cannot ignore the luminance of the rest of the frame, because it is also important, especially when there is no other light source during the presentation of the movies or when the light in the room is very dim.  Figure 8 shows the changes in the pupil diameter as determined using both of the abovementioned methods. The correlation between the pupil diameter determined by the average luminance of the entire frame and using the luminance in the neighbourhood of the gaze position was 0.87. A very strong correlation indicates that both methods work similarly. For each participant, the correlation of the mean pupil diameter for each movie, related to one emotion with the average value of the movie luminance (i.e., without removing the influence of luminance), was also calculated. The same correlations were calculated after removing the influence of luminance (by using the average luminance of the entire frame). The median of correlation values calculated for all participants was −0.84 in the first case and −0.30 in the second case. The large difference in these values indicates that the method proposed by us for removing the effect of luminance is sufficiently effective.

**Figure 9:**
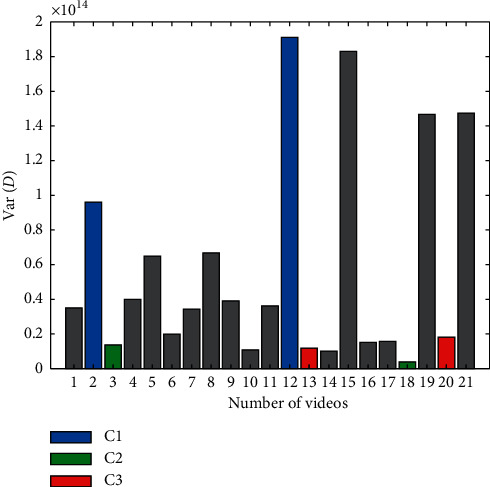
Movie dynamics index.

In [28, 30, 38], attempts were made to use eye-tracking to recognize emotions caused by movies. However, these works did not consider the possible impact of movie dynamics on eye-tracking features. The features discussed in this article, which are associated with eye movements, may also be related to factors other than emotions. Hence, we tested the relationship of the eye-tracking features with the dynamics of the presented movies. Movies can be characterized by frequent or rare scene changes and moving or static objects. Eye movements such as fixation and saccades depend largely on the dynamics of the movie scene. This impact cannot be ignored when analyzing the signal for emotion recognition. We calculated a *D* index describing the dynamics of the video clip

(5) $$\begin{array}{c} D_{i}=\sum_{j}^{N}\sum_{k}^{M}\left(F_{i+1}-F_{i}\right), \end{array}$$

where *F* is the matrix representing video frame of size *N* × *M* in grayscale and *i* is the frame number.  Figure 9 depicts the variation of the *D* index for each of the movies shown. Thus, each movie was rated for the speed at which scenes or objects changed between frames.

**Figure 10:**
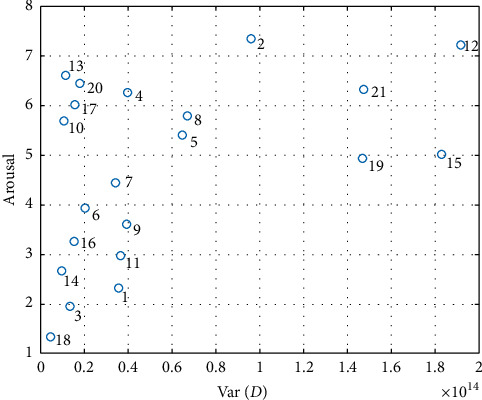
Dependence of arousal parameter on the movie dynamics.

The obtained classification results indicate that the most difficult emotions to distinguish were negative, highly stimulating emotions from neutral, low stimulating ones (class C2 vs. C3). These two classes contain two pairs of movies: #3 and #18 with #13 and #20. According to the calculated dynamics index, these movies are characterized by small dynamics. Their dynamics are definitely smaller than those of movies #2 and #12 in class C1. The above tendency may indicate that the classification result, to some extent, also depends on movie dynamics and not just only on its emotional content. The distinction between the two classes of movies of similar dynamics seems to be more difficult than that between the movies of different dynamics. To test the effect of video dynamics on the obtained results, a correlation of the coefficient *D* with the used features, averaged for all participants, was calculated. The correlations are shown in Table 10. We obtained the highest values of the average correlation (≥0.4) for the following features: #1—number of fixations, #13—skewness of the saccade durations, and #15—average pupil diameter. The ANOVA test showed that the average pupil diameter and skewness of the saccade durations were identified as statistically significant for emotion recognition (Table 8). This may indicate a relationship between the effectiveness of emotion recognition and movie dynamics. The correlation between the participants' ratings of movies used in the experiment with their dynamics was 0.45 for the arousal. These values are statistically significant and may indicate that stimulating videos are characterized by high dynamics (#2, #12, #15, #19, and #21) and nonstimulating videos by low dynamics (#1, #3, #14, and #18). Dependence of arousal parameter on the movie dynamics is shown in Figure 10.

Our database contains only a few specific movies that are stimulating and not dynamic. These are, for example, movies showing finger amputation (movie #20) or tooth extraction (movie #10). In these movies, the camera does not change its position, and the scenes are static. Therefore, the dynamic index for these movies is low. These movies are rated as stimulating because they cause strong negative emotions related to disgust. Movies #4 and #17 depict horror scenes. After a few static scenes (low dynamics), terrible characters appear in these movies, thereby causing high arousal.

The weakness of the work is the possible impact of movie dynamics on the results achieved. However, when recognizing the emotions evoked by movies with similar dynamics, a satisfactory classification accuracy of 78% was achieved using the LDA classifier. In the future, it is worth conducting additional research on the impact of movie dynamics.

The obtained results were compared with the results from other publications in which eye-tracking was used. However, it should be borne in mind that accurate comparison is difficult because each study used a different way of evoking emotions, a different set of eye-tracking features, and different research group. The comparison is presented in Table 11. The conducted comparison indicates that the results obtained by us are slightly better than those in similar works. The choice of a narrow group taking part in the experiment (young men) may affect the result. It is possible that, in such a coherent group of people, emotions were evoked more precisely.

## 5. Conclusions

The classification results confirm the ability of recognizing emotions using eye movement features and pupil diameter. The use of an eye-tracking requires, however, the elimination of factors that may affect this classification. These factors include the effect of luminance on changes in the pupil diameter. It should be ensured that the lighting conditions remain the same throughout the experiment. The influence of luminance of the presented materials should also be compensated. This could be achieved by implementing appropriate methods, such as regression or principal component analysis.

Another factor that should be considered is the dynamics of the presented material. It seems that the dynamics of the movie can affect the accuracy of the emotion classification using the eye-tracking features. On the other hand, the dynamics of movie is related in some way to felt emotions. Research shows that high-dynamic movies have a stimulating effect. When recognizing the emotions evoked by movies with similar dynamics, a satisfactory classification accuracy of 78% using LDA was achieved. During recognition of three classes of emotions, we obtained the maximum classification accuracy of 80%. It is worth emphasizing that all results were obtained using the leave-one-subject-out validation method. This implies that the presented user-independent method of emotion classification, based on eye-tracking features, can be successfully used in practice.

## Figures and Tables

**Table 1:** Evaluation of the selected movies.

Class	Video numbers	Duration (s)	Arousal		Valence	
			Mean	Median	Mean	Median

C1	2	10	7.3	8	8.1	9
	12	13	7.2	7.5	7.9	8.5

C2	3	5	1.9	1	5.6	5
	18	10	1.3	1	4.8	5

C3	13	10	6.6	6.5	2.8	2
	20	10	6.4	6	3.3	2.5

**Table 2:** List of calculated features.

Signal type	Features	No.
Fixation	Number of fixations	1
	Overall fixation vector	2

Fixation duration	Mean	3
	Variance	4
	Skewness	5
	Kurtosis	6

Saccade amplitude	Mean	7
	Variance	8
	Skewness	9
	Kurtosis	10

Saccade duration	Mean	11
	Variance	12
	Skewness	13
	Kurtosis	14

Pupil diameter	Mean	15
	Variance	16
	Skewness	17
	Kurtosis	18

**Table 3:** Classification accuracies.

Classes	SVM	LDA	*k*-NN	Mean
C1 vs. C2 vs. C3	0.80	0.73	0.65	0.73
C1 vs. C2	0.92	0.85	0.86	0.88
C1 vs. C3	0.83	0.84	0.75	0.81
C2 vs. C3	0.75	0.78	0.70	0.74

**Table 4:** Confusion matrix for SVM classifier.

Predicted classes	True classes		
	C1	C2	C3
C1	45	3	7
C2	1	42	10
C3	4	5	33

**Table 5:** Confusion matrix for LDA classifier.

Predicted classes	True classes		
	C1	C2	C3
C1	41	6	7
C2	1	34	5
C3	8	10	38

**Table 6:** Confusion matrix for *k-*NN classifier.

Predicted classes	True classes		
	C1	C2	C3
C1	48	13	20
C2	0	33	13
C3	2	4	17

**Table 7:** Precision, recall, specificity, and F1-score for all classifiers.

Classes	SVM				LDA				*k-*NN			
	P	R	S	F1	P	R	S	F1	P	R	S	F1
C1	0.82	0.90	0.90	0.86	0.76	0.82	0.87	0.79	0.59	0.96	0.67	0.73
C2	0.79	0.84	0.89	0.81	0.85	0.68	0.94	0.76	0.72	0.66	0.87	0.69
C3	0.79	0.66	0.91	0.72	0.68	0.76	0.82	0.72	0.74	0.34	0.94	0.47
Mean	0.80	0.80	0.90	0.80	0.76	0.75	0.88	0.76	0.68	0.65	0.83	0.63

**Table 8:** Results of ANOVA and Bonferroni post-hoc tests.

Feature	ANOVA		Bonferroni multiple comparison test		
	*p*	*p* < 0.05	C1 vs. C2 *p*	C1 vs. C3 *p*	C2 vs. C3 *p*
Number of fixations	0.32	−	1.00	1.00	0.40
Overall fixation vector	0.64	−	1.00	1.00	1.00
Average duration of fixation	**0.01**	+	1.00	**0.01**	**0.02**
Variance of fixation duration	0.06	−	1.00	0.06	0.43
Skewness of fixation duration	**0.00**	+	**0.01**	1.00	**0.00**
Kurtosis of fixation duration	**0.00**	+	**0.01**	1.00	**0.01**
Average saccade amplitude	**0.00**	+	**0.00**	0.65	**0.00**
Variance of the saccade amplitudes	**0.00**	+	**0.00**	**0.02**	0.96
Skewness of the saccade amplitudes	**0.00**	+	**0.00**	**0.01**	1.00
Kurtosis of the saccade amplitudes	**0.00**	+	**0.00**	**0.00**	1.00
Average duration of saccades	0.86	−	1.00	1.00	1.00
Variance of saccade durations	0.56	−	1.00	0.89	1.00
Skewness of saccade durations	**0.00**	+	**0.00**	**0.01**	0.99
Kurtosis of saccade durations	**0.00**	+	**0.00**	**0.00**	0.71
Average pupil diameter	**0.00**	+	**0.00**	**0.00**	0.35
Variance of the pupil diameter	**0.04**	+	0.29	1.00	**0.03**
Skewness of the pupil diameter	0.09	−	1.00	0.23	0.12
Kurtosis of the pupil diameter	0.05	−	0.51	**0.04**	0.82

**Table 9:** Mean values and standard deviations of all features for three classes of emotions.

No.	Feature	C1		C2		C3	
		Mean	Std.	Mean	Std.	Mean	Std.
1	Number of fixations	−0.33	0.50	−0.29	0.88	0.17	1.08
2	Overall fixation vector	−0.31	0.47	−0.16	1.05	0.09	0.91
3	Average duration of fixation	0.11	0.95	−0.50	0.85	0.18	1.11
4	Variance of fixation duration	0.17	1.04	−0.42	0.63	0.15	1.14
5	Skewness of fixation duration	0.18	0.76	0.03	1.23	−0.13	1.00
6	Kurtosis of fixation duration	−0.31	0.48	1.00	1.00	−0.11	0.90
7	Average saccade amplitude	−0.33	0.51	0.48	0.88	0.27	1.52
8	Variance of the saccade amplitudes	−0.37	0.60	0.17	0.96	0.17	0.88
9	Skewness of the saccade amplitudes	−0.44	0.59	0.24	0.85	0.11	0.86
10	Kurtosis of the saccade amplitudes	0.12	0.93	0.09	1.26	0.01	1.07
11	Average duration of saccades	0.15	1.07	0.08	1.22	−0.08	0.97
12	Variance of saccade durations	0.31	0.89	−0.36	0.69	−0.20	0.83
13	Skewness of saccade durations	0.33	1.03	−0.43	0.54	−0.24	0.77
14	Kurtosis of saccade durations	0.20	0.74	0.04	0.93	0.17	0.98
15	Average pupil diameter	0.85	0.83	−0.73	1.17	−0.43	0.83
16	Variance of the pupil diameter	−0.04	0.68	−0.31	0.69	0.11	1.04
17	Skewness of the pupil diameter	−0.06	0.66	−0.01	0.72	−0.35	1.06
18	Kurtosis of the pupil diameter	0.09	0.66	−0.13	0.81	−0.31	0.89

**Table 10:** Correlation of the coefficient *D* with the used features averaged for all participants.

No.	Feature	Movie dynamics
1	Number of fixations	0.43
2	Overall fixation vector	−0.34
3	Average duration of fixation	−0.32
4	Variation of fixation durations	−0.38
5	Skewness of fixation durations	0.28
6	Kurtosis of fixation durations	0.30
7	Average amplitude of the saccades	−0.35
8	Variation of saccade amplitudes	−0.30
9	Skewness of saccade amplitudes	−0.10
10	Kurtosis of saccade amplitudes	−0.17
11	Average duration of saccades	0.07
12	Variance of the saccade durations	0.10
13	Skewness of the saccade durations	0.40
14	Kurtosis of the saccade durations	0.39
15	Average pupil diameter	0.62
16	Pupil diameter variance	0.13
17	Pupil diameter skewness	0.16
18	Pupil diameter kurtosis	0.25

**Table 11:** Comparison of our results with other studies.

Works	Classes	User-independent method	Accuracy (%)
Our research	3 (high arousal and high valence, high arousal and low valence, low arousal and moderate valence)	+	80.00
Soleymani et al. [57]	3 (calm, medium aroused, and activated)	+	71.10
Soleymani et al. [57]	3 (unpleasant, neutral, and pleasant)	+	66.60
Zheng et al. [30]	4 (sad, feeling fear, happy, and neutral)	−	67.82
Lu et al. [28]	3 (positive, neutral, and negative)	−	77.80
Soleymani et al. [38]	3 (calm, medium aroused, and excited)	+	63.50
Soleymani et al. [38]	3 (unpleasant, neutral, and pleasant)	+	68.80
Zhao et al. [31]	5 (happy, sad, feeling fear, disgusted, and neutral)	−	59.81

## Data Availability

The data used to support the findings of this study are available from the corresponding author upon request.
